# Radiological Outcome of Middle Meningeal Artery Embolization in Relation to Chronic Subdural Hematoma Cause and Architecture

**DOI:** 10.3390/brainsci14111097

**Published:** 2024-10-30

**Authors:** Ahmed Abdelghafar, Andrew Falzon, Eef J. Hendriks, Ivan Radovanovic, Hugo Andrade, Joanna D. Schaafsma, Pascal J. Mosimann

**Affiliations:** 1 Division of Neuroradiology, Joint Department of Medical Imaging, Toronto Western Hospital, University Health Network, Toronto, ON M5T 2S8, Canada; 2 Atkinson Morley Regional Neuroscience Centre, St George’s University Hospital, Tooting, London SW17 0QT, UK; 3 Department of Medical Imaging, University of Toronto, Toronto, ON M5T 1W7, Canada; 4 Division of Neurosurgery, Toronto Western Hospital, Toronto, ON M5T 2S8, Canada; 5 Sprott Department of Surgery, University of Toronto, Toronto, ON M5G 2C4, Canada; 6 Department of Neurology, University of Toronto, Toronto, ON M5S 3H2, Canada

**Keywords:** middle meningeal artery embolization, chronic subdural hematoma, chronic subdural hematoma architecture, chronic subdural hematoma cause

## Abstract

**Background/Objectives**: MMAE (middle meningeal artery embolization) has emerged as a potential effective treatment for cSDH (chronic subdural hematoma). In this study, MMAE efficiency with regards to cSDH cause and architecture was explored. The comparability of cSDH thickness and volume as parameters for cSDH pre- and post-MMAE assessment was also analyzed. **Methods**: In this retrospective cohort study, 52 consecutive cSDH patients treated with MMAE in a single tertiary center were included. The cohort was divided into two group pairs pertaining to cSDH cause (spontaneous or traumatic) and cSDH architecture (non-mature or mature). The radiological outcome was compared in each group before and after MMAE and between each group pair using CT imaging. A correlation analysis between cSDH thickness and volume before and after MMAE was also performed. **Results**: A statistically significant positive linear association between cSDH thickness and volume at admission and at each follow-up interval (1–3, 3–6, 6–12 months) was noticed. cSDH thickness and volume reduction in each group was statistically significant, except for a traumatic cSDH volume reduction at 6–12 months. There was no statistically significant difference between each group pair in the cSDH thickness and volume reduction difference at all the follow-up intervals. **Conclusions**: A comparable efficiency of MMAE may be achieved in non-mature and mature as well as in spontaneous and traumatic cSDH, with an advantage for spontaneous cSDH at 6–12 months follow-up compared to traumatic cSDH. Traumatic cSDH may require a relatively long-term follow-up post-MMAE. cSDH thickness and volume, as parameters for pre- and post-MMAE cSDH evaluation, appear similar.

## 1. Introduction

cSDH (chronic subdural hematoma) is a relatively common condition associated with increased morbidity and mortality [1,2]. It has an increased propensity to persist or grow, if left untreated, due to a potentially ongoing neurovascular inflammatory cascade [3,4,5]. cSDH growth may be associated with poor functional outcome and high mortality rates [2,6]. The appropriate diagnosis and management of cSDH are therefore important to hinder its progression.

cSDH may be managed conservatively, if grossly asymptomatic. It may be managed with surgical drainage via a twist drill, craniotomy, burr hole craniotomy, or mini craniotomy in those who are symptomatic [7,8,9].

MMAE (middle meningeal artery embolization) is increasingly considered for cSDH treatment [10,11,12,13]. It was postulated that MMAE interrupts the blood supply of the neovascular membrane of cSDH, thought to be responsible for cSDH formation and growth [14,15]. Multiple studies have demonstrated a favorable cSDH outcome after MMAE [16,17].

The study goals are to analyze and compare MMAE efficiency in specific cohort groups with regards to cSDH cause and architecture and to assess the similarity between cSDH thickness and volume as radiological parameters for cSDH evaluation before and after MMAE.

## 2. Materials and Methods

This retrospective cohort study included 52 consecutive cases, diagnosed with cSDH and treated with MMAE between January 2020 and December 2023 in a single tertiary center.

The STROBE checklist was used throughout.

The inclusion criteria were as follows:

1. Patients diagnosed with cSDH, treated with MMAE and followed up for at least 1 month;
2. Available clinical data, procedural details, pre-procedural and follow-up CT scans.

Exclusion criteria included the following:

1. Insufficient DICOM (Digital Imaging and Communications in Medicine);
2. Post-MMAE surgical evacuation.

Post-MMAE surgical evacuation was thought to possibly overestimate or underestimate cSDH measurements at follow-up.

Clinical and imaging data were extracted from the center’s research database and electronic medical records.

### 2.1. MMAE Procedural Details

MMAE was performed in a sterile condition in a biplane neuroangiography suite. The procedure was performed either under general anesthesia or conscious sedation and the arterial access was either through the right femoral or the right radial artery.

Selective catheterization was carried out using a coaxial or tri-axial technique. The anatomical origin of the ipsilateral ophthalmic artery was confirmed through CCA or ICA angiography. Further anatomy was confirmed through selective microcatheter angiography of the middle meningeal artery trunk, frontal, and parietal branches.

The embolization technique entailed either PVA (polyvinyl alcohol) particles, PVA particles–microcoils, liquid embolic agents (Onyx), microcoils, or Onyx–microcoils. The technique used was determined based on collateralization and anatomy.

### 2.2. Imaging Assessment

CT imaging was performed before MMAE and at follow-up, in compliance with the center’s guidelines for cSDH patient management.

cSDH volume was measured using the ABC/2 method [18]. The cSDH thickness was the same as that used in calculating cSDH volume.

In cases with pre-MMAE surgical evacuation, the last CT scan before MMAE and after surgical evacuation was considered for hematoma thickness and volume assessment at admission.

### 2.3. Statistics

Statistical analysis was carried out using IBM SPSS statistics 29.0.2.0. Continuous variables were described with mean, median, standard deviation, and/or confidence interval.

Categorical variables were described with frequency and/or percentage.

The correlation between the cSDH thickness and the volumes at admission and at each follow-up interval was evaluated. Shapiro–Wilk test was performed for all variables. Correlation test results were described with Pearson’s correlation coefficient (r) for direction and strength and *p*-value for significance of the association.

The cohort was divided into 2 group pairs in relation to cSDH cause and architecture.

cSDH is defined as hematoma collection in the subdural space with outer and inner membranes [19].

cSDH cause is either spontaneous or traumatic [20].

cSDH architecture at admission is either type 1 homogenous/laminar (non-mature cSDH) or type 2 separated/trabecular (mature cSDH) [21].

In each group, pre- and post-MMAE cSDH thickness and volume were compared. Paired *t*-test and Wilcoxon signed-rank test were used.

Post-MMAE radiological outcome was defined as cSDH thickness/volume difference, which is the difference between cSDH thickness/volume at follow-up and at admission. Furthermore, 1–3, 3–6, 6–12 months’ follow-up intervals were included.

cSDH thickness/volume difference was compared between each group pair at each follow-up interval. Univariate analysis was conducted with an independent *t*-test, Mann–Whitney U test, ANCOVA, and/or generalized linear model (GLM).

The Shapiro–Wilk test was performed for the compared variables in each group to assess normal distribution. Levene’s test was performed to evaluate the equality of variance for the variables compared between each group pair.

The following possible covariates were compared between groups: age, sex, antiplatelet therapy, anticoagulant therapy, hepatic comorbidity, alcohol intake, dementia, previous surgical evacuation, MMAE technique, and PVA particle size. Hematoma architecture type was additionally considered for cSDH cause group comparison.

Current antiplatelets and/or anticoagulant therapy have been defined as concomitant treatment by antiplatelet or anticoagulant at admission. Hepatic comorbidity has been defined as the diagnosis of any hepatic digestive, synthetic, metabolic or excretory dysfunction at admission [22]. Current alcohol intake was defined as lifetime drinking of ≥12 drinks and an average of >7 drinks per week for women and >14 drinks per week for men in the previous year to admission [23]. Dementia was defined as a decline in memory, executive functions, and behavioral changes with functional impairment [24].

The comparison between each group pair was performed with a Chi-Squared test for the categorical covariates, and the independent *t*-test and/or Mann–Whitney U test for the continuous covariate.

The Shapiro–Wilk test and Levene’s test were carried out for the continuous covariate.

Statistical significance was defined as *p*-value < 0.05.

### 2.4. Ethics, Informed Consent and Confidentiality

Research ethics board approval was obtained (ID 24-5029). Written consent was waived due to the retrospective type of study. Data were anonymized.

## 3. Results

### 3.1. cSDH Volume and Thickness Correlation

The association between cSDH thickness and volume at admission was evaluated. This was also evaluated at each follow-up interval.

Pearson’s correlation yielded a statistically significant strong positive linear association (*p* < 0.001, r = 1) between the thickness and volume at admission and at each follow-up interval.

All compared variables were normally distributed.

### 3.2. cSDH Cause Groups

The cohort was divided into 21 spontaneous cSDH cases (group 1) and 31 traumatic cSDH cases (group 2).

The mean age in the first group was 71, with a range of 62 and standard deviation of 15. Up to 7/21 (33.3%) were females and 14/21 (66.7%) were males. The embolization technique used was PVA in 12 cases, PVA–microcoils in 7 cases, Onyx in 1 case, and microcoils in 1 case.

In the second group, the mean age was 73, while the age range was 37 with a standard deviation of 10. Up to 6/31 (19.4%) cases were females, and 25/31 (80.6%) cases were males. The embolization technique used was PVA in 12 cases, PVA–microcoils in 17 cases, Onyx in 1 case, and Onyx–microcoils in 1 case.

Hepatic disease (*p* = 0.022), antiplatelets (*p* = 0.01), and particle type (*p* = 0.005) showed statistically significant difference between the groups.

The results showed statistically significant lower values of cSDH thickness and volume at each follow-up interval for each group compared to the admission values, except for the traumatic cSDH volume at 6–12 months. The traumatic cSDH volume reduction at 6–12 months was not statistically significant (Table 1a,b).

The mean thickness and volume reduction difference at 1–3 and 3–6 months in the traumatic cSDH was higher than the spontaneous cSDH. However, this was higher at 6–12 months in the spontaneous cSDH compared to the traumatic cSDH.

The thickness/volume reduction differences between the group pairs were statistically significant at 3–6 months.

The thickness/volume reduction difference between the groups was not statistically significant at all the follow-up intervals after the correction for covariates (Table 2).

### 3.3. cSDH Architecture Groups

**Figure 1:**
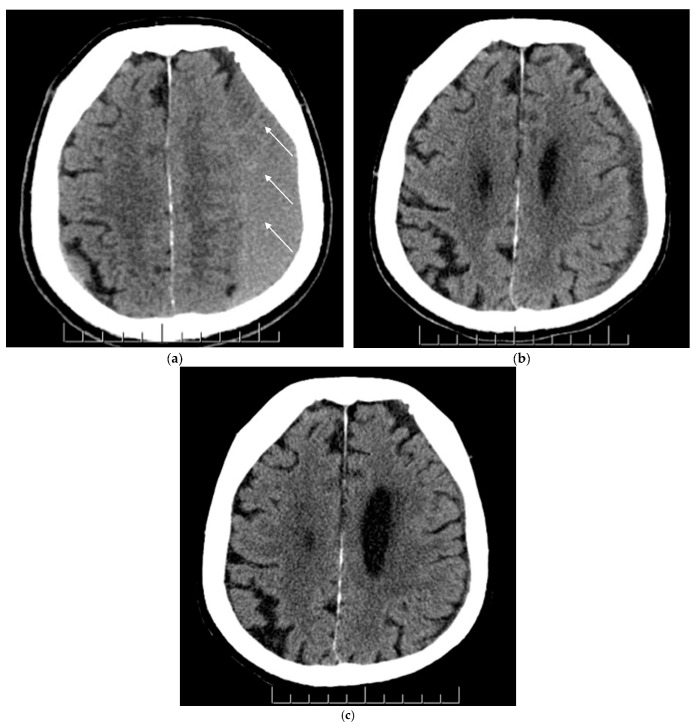
(**a**–**c**) Axial view of non-contrast CT head scans before and after MMAE (middle meningeal artery embolization) for the same patient. Figure parts a, b and c represent chronic subdural hematoma (cSDH) pre-MMAE (baseline), at 1–3 months and 3–6 months post-MMAE, respectively. (**a**) A non-mature mixed cSDH was noticed at baseline on the left cerebral hemisphere with sulcal effacement, obliteration of the left lateral ventricle and slight midline shift to the right. The white arrows demonstrate the mixed cSDH nature; (**b**) Improvement of the cSDH and mass effect was noticed in 6-week CT scan post-MMAE; (**c**) A near-complete resolution of cSDH and mass effect was noticed in 5-month CT scan.

A total of 23 cases had type 1 cSDH, and 29 had type 2 at admission (Figure 1).

In the type 1 group, the mean age was 74 with a range of 34 and standard deviation of 9.

Up to 8/23 cases (34.8%) were females and 15/23 (65.2%) were males. A total of 8 cases had PVA particles, while 15 had combined PVA–microcoils.

In type 2 group, the mean age was 71 with a standard deviation of 14 and a range of 64. Approximately 5/29 cases (17.2%) were females and 24/29 (82.8%) were males.

The embolization results for type 2 group were as follows: 16 PVA, 9 PVA–microcoils, 2 Onyx, 1 with microcoils, and 1 combined Onyx–microcoils.

None of the covariates showed a statistically significant difference between the groups.

There were statistically significant lower values of cSDH thickness/volume at each follow-up interval for each group compared to the admission values (Table 1a,b).

The mean thickness and volume reduction difference in the type 1 hematoma was higher than the type 2 at all follow-up intervals.

The thickness/volume reduction difference between groups was not statistically significant at all the follow-up intervals (Table 3).

## 4. Discussion

### 4.1. Imaging Parameters for Post-MMAE cSDH Change Assessment

The cSDH volume and thickness are frequently used parameters in assessing cSDH size [25,26]. Multiple techniques for thickness and volume measurements have been proposed [27,28].

The initial evaluation of cSDH thickness and volume appeared effective in cSDH prognosis prediction after surgery [29,30]. The question was whether both parameters were comparable in evaluating cSDH change after MMAE.

The results showed a very strong positive correlation between the cSDH thickness and volume before and after the MMAE. cSDH thickness and volume appear to be comparable parameters in evaluating the cSDH radiological outcomes after the MMAE.

### 4.2. cSDH Cause

cSDH develops as a result of chronic inflammatory neovascularization bleeding within the hematoma membrane in the subdural space [31]. Trauma and spontaneous bleeding have been described as potential causes that may provoke this inflammatory and neoangiogenic process [32,33]. cSDH is not necessarily preceded by an acute subdural hematoma or traumatic event [34,35].

The results suggest that the cSDH volume/thickness reduction in each group was statistically significant, except for the cSDH volume reduction at 6–12 months in the traumatic cSDH group (Table 1a,b). Additionally, the cSDH volume/thickness reduction difference between groups was not statistically significant (Table 2).

MMAE appears to have similar efficiency in hematoma reduction when comparing both groups. Traumatic cSDH may need a relatively long-term follow-up to monitor the radiological outcomes (Table 1a,b and Table 2).

### 4.3. cSDH Architecture

Different hematoma architecture types were suggested, including homogenous, laminar, separated, and trabecular [21,36,37]. cSDH trajectory was thought to initially start with homogenous/laminar types and then develop into mature separated/trabecular types [21].

The initial cSDH types were associated with a relatively faster rate of hematoma volume reduction compared to the mature cSDH types after MMAE [38].

For each group, the results showed a significant cSDH thickness and volume reduction post-MMAE (Table 1a,b). When comparing both groups, cSDH thickness and volume reduction difference was higher in the type 1 hematoma compared to type 2 at all follow-up intervals. This difference was not statistically significant (Table 3).

Both the non-mature and mature cSDH types appeared to have a comparable efficient radiological outcome after the MMAE (Table 1a,b and Table 3).

### 4.4. Limitations

The retrospective nature of the study may be associated with recall bias. We therefore assessed the clinical information in detail and in variable encounters.

To ensure accuracy and tackle potential bias, double blinded measurements were performed by two experienced researchers for imaging data evaluation. A review from an experienced independent reviewer was added in case of any discrepancy.

## 5. Conclusions

MMAE appears similarly efficient in cSDH thickness and volume reduction pertaining to both non-mature and mature hematoma architectures. It appears to have a comparable, efficient radiological outcome for both spontaneous and traumatic cSDH, with an edge for the spontaneous type on a long-term follow-up. Traumatic cSDH may require a relatively long follow-up time to assess the radiological outcomes. cSDH thickness and volume appear to be comparable radiological parameters in the evaluation of cSDH before and after MMAE.

## Figures and Tables

**Table 1:** (**a**) Comparison of cSDH (chronic subdural hematoma) thickness/volume before and after MMAE (middle meningeal artery embolization) in relation to cSDH cause and architecture type, using paired *t*-test. *p* < 0.05 was considered statistically significant. (**b**) Comparison of cSDH thickness/volume before and after MMAE with regards to cSDH cause and architecture type, using Wilcoxon signed-rank test. *p* < 0.05 was considered statistically significant.

**(a)**				
**Thickness (T) and Volume (V) at** **Admission (0) and at Interval Follow-Up**	***T*-Test Significance (*p* < 0.05)**			
	**cSDH Cause**		**cSDH Architecture**	
	**Spontaneous**	**Traumatic**	**Non-Mature**	**Mature**
T 0—at 1–3 months (mm)	<0.001	<0.001	<0.001	<0.001
V 0—at 1–3 months (cm^3^)	<0.001	<0.001	<0.001	<0.001
T 0—at 3–6 months (mm)	<0.001	<0.001	<0.001	<0.001
V 0—at 3–6 months (cm^3^)	0.001	<0.001	<0.001	<0.001
T 0—at 6–12 months (mm)	<0.001	<0.001	<0.001	0.025
V 0—at 6–12 months (cm^3^)	<0.001	<0.001	<0.001	0.003
**(b)**				
**Thickness (T) and Volume (V) at** **Admission (0) and at Interval Follow-Up**	**Wilcoxon Signed-Rank Test Significance (*p* < 0.05)**			
	**cSDH Cause**		**cSDH Architecture**	
	**Spontaneous**	**Traumatic**	**Non-Mature**	**Mature**
T 0—at 1–3 months (mm)	<0.001	<0.001	<0.001	<0.001
V 0—at 1–3 months (cm^3^)	<0.001	<0.001	<0.001	<0.001
T 0—at 3–6 months (mm)	0.012	<0.001	0.008	0.001
V 0—at 3–6 months (cm^3^)	0.012	<0.001	0.008	0.001
T 0—at 6–12 months (mm)	0.002	0.068	0.008	0.018
V 0—at 6–12 months (cm^3^)	0.002	0.068	0.008	0.018

**Table 2:** Comparison of thickness and volume reduction differences at follow-up between spontaneous and traumatic cSDH groups. *p* < 0.05 was considered statistically significant.

Spontaneous–Traumatic cSDH Groups Differences	Mean Difference	95% CI		Significance (*p* < 0.05)			
		Lower	Upper	*T*-Test	MWU *	ANCOVA	GLM *
T Difference at 1–3 Months (mm) *	−0.37	−5.13	4.40	0.438	0.910	0.145	0.119
V Difference at 1–3 Months (cm^3^) *	−1.68	−32.37	29.01	0.456	0.860	0.187	0.172
T Difference at 3–6 months (mm)	−7.32	−13	−1.63	0.007	0.044	0.329	0.536
V Difference at 3–6 Months (cm^3^)	−34.21	−74.07	5.63	0.044	0.048	0.559	0.670
T Difference at 6–12 Months (mm)	0.67	−8.84	10.18	0.44	0.626	0.253	0.138
V Difference at 6–12 Months (cm^3^)	18.46	−37.88	74.8	0.247	0.716	0.467	0.354

* GLM, generalized linear model MWU; Mann-Whitney U test; T, cSDH thickness; V, cSDH volume.

**Table 3:** Hematoma type 1 and 2 groups thickness and volume reduction differences comparison at follow-up. *p* < 0.05 was considered statistically significant.

Hematoma Type 1– Type 2 Groups Differences	Mean Difference	95% CI		Significance (*p* < 0.05)	
		Lower	Upper	*T*-Test	MWU *
T Difference at 1–3 Months (mm) *	0.1	−4.52	4.76	0.479	0.816
V Difference at 1–3 Months (cm^3^) *	2.8	−27.06	32.68	0.425	0.864
T Difference at 3–6 Months (mm)	4	−2.97	11.01	0.122	0.198
V Difference at 3–6 Months (cm^3^)	23	−17.06	63.06	0.122	0.356
T Difference at 6–12 Months (mm)	5.7	−1.92	13.35	0.065	0.089
V Difference at 6–12 Months (cm^3^)	7.7	−42.13	57.56	0.372	0.368

* MWU, Mann–Whitney U test; T, cSDH thickness; V, cSDH volume.

## Data Availability

The data presented in this study are available on request from the corresponding authors due to ethical restrictions.

## References

[1] Miranda L.B., Braxton E., Hobbs J., Quigley M.R. (2011). Chronic Subdural Hematoma in the Elderly: Not a Benign Disease: Clinical Article. J. Neurosurg..

[2] Feghali J., Yang W., Huang J. (2020). Updates in Chronic Subdural Hematoma: Epidemiology, Etiology, Pathogenesis, Treatment, and Outcome. World Neurosurg..

[3] Weigel R., Schilling L., Schmiedek P. (2001). Specific Pattern of Growth Factor Distribution in Chronic Subdural Hematoma (CSH): Evidence for an Angiogenic Disease. Acta Neurochir..

[4] Ducruet A.F., Grobelny B.T., Zacharia B.E., Hickman Z.L., DeRosa P.L., Anderson K., Sussman E., Carpenter A., Connolly E.S. (2012). The Surgical Management of Chronic Subdural Hematoma. Neurosurg. Rev..

[5] Tamura R., Sato M., Yoshida K., Toda M. (2021). History and Current Progress of Chronic Subdural Hematoma. J. Neurol. Sci..

[6] Yang W., Huang J. (2017). Chronic Subdural Hematoma. Neurosurg. Clin. N. Am..

[7] Kolias A.G., Chari A., Santarius T., Hutchinson P.J. (2014). Chronic Subdural Haematoma: Modern Management and Emerging Therapies. Nat. Rev. Neurol..

[8] Blaauw J., Jacobs B., Den Hertog H.M., Van Der Gaag N.A., Jellema K., Dammers R., Lingsma H.F., Naalt J.V.D., Kho K.H., Groen R.J.M. (2020). Neurosurgical and Perioperative Management of Chronic Subdural Hematoma. Front. Neurol..

[9] Foppen M., Bandral H.V., Slot K.-A.M., Vandertop W.P., Verbaan D. (2023). Success of Conservative Therapy for Chronic Subdural Hematoma Patients: A Systematic Review. Front. Neurol..

[10] Link T.W., Rapoport B.I., Paine S.M., Kamel H., Knopman J. (2018). Middle Meningeal Artery Embolization for Chronic Subdural Hematoma: Endovascular Technique and Radiographic Findings. Interv. Neuroradiol..

[11] Srivatsan A., Mohanty A., Nascimento F.A., Hafeez M.U., Srinivasan V.M., Thomas A., Chen S.R., Johnson J.N., Kan P. (2019). Middle Meningeal Artery Embolization for Chronic Subdural Hematoma: Meta-Analysis and Systematic Review. World Neurosurg..

[12] Fiorella D., Arthur A.S. (2019). Middle Meningeal Artery Embolization for the Management of Chronic Subdural Hematoma. J. Neurointerv. Surg..

[13] Haldrup M., Ketharanathan B., Debrabant B., Schwartz O.S., Mikkelsen R., Fugleholm K., Poulsen F.R., Jensen T.S.R., Thaarup L.V., Bergholt B. (2020). Embolization of the Middle Meningeal Artery in Patients with Chronic Subdural Hematoma—A Systematic Review and Meta-Analysis. Acta Neurochir..

[14] Ishihara H., Ishihara S., Kohyama S., Yamane F., Ogawa M., Sato A., Matsutani M. (2007). Experience in Endovascular Treatment of Recurrent Chronic Subdural Hematoma. Interv. Neuroradiol..

[15] Ban S.P., Hwang G., Byoun H.S., Kim T., Lee S.U., Bang J.S., Han J.H., Kim C.-Y., Kwon O.-K., Oh C.W. (2018). Middle Meningeal Artery Embolization for Chronic Subdural Hematoma. Radiology.

[16] Catapano J.S., Ducruet A.F., Nguyen C.L., Baranoski J.F., Cole T.S., Majmundar N., Wilkinson D.A., Fredrickson V.L., Cavalcanti D.D., Albuquerque F.C. (2021). Middle Meningeal Artery Embolization for Chronic Subdural Hematoma: An Institutional Technical Analysis. J. Neurointerv. Surg..

[17] Ironside N., Nguyen C., Do Q., Ugiliweneza B., Chen C.-J., Sieg E.P., James R.F., Ding D. (2021). Middle Meningeal Artery Embolization for Chronic Subdural Hematoma: A Systematic Review and Meta-Analysis. J. Neurointerv. Surg..

[18] Gebel J.M., Sila C.A., Sloan M.A., Granger C.B., Weisenberger J.P., Green C.L., Topol E.J., Mahaffey K.W. (1998). Comparison of the ABC/2 Estimation Technique to Computer-Assisted Volumetric Analysis of Intraparenchymal and Subdural Hematomas Complicating the GUSTO-1 Trial. Stroke.

[19] Robinson R.G. (1984). Chronic Subdural Hematoma: Surgical Management in 133 Patients. J. Neurosurg..

[20] Lee K.-S. (2004). ReviewNatural History of Chronic Subdural Haematoma. Brain Inj..

[21] Nakaguchi H., Tanishima T., Yoshimasu N. (2001). Factors in the Natural History of Chronic Subdural Hematomas That Influence Their Postoperative Recurrence. J. Neurosurg..

[22] Tennant B.C. (1997). Hepatic Function. Clinical Biochemistry of Domestic Animals.

[23] Schoenborn C.A., Adams P.E. (2010). Health Behaviors of Adults: United States, 2005–2007. Vital Health Statistics.

[24] World Health Organization (1992). The ICD-10 Classification of Mental and Behavioural Disorders: Clinical Descriptions and Diagnostic Guidelines.

[25] Omura Y., Ishiguro T. (2023). Middle Meningeal Artery Embolization for Chronic Subdural Hematoma: A Systematic Review. Front. Neurol..

[26] Levitt M.R., Hirsch J.A., Chen M. (2024). Middle Meningeal Artery Embolization for Chronic Subdural Hematoma: An Effective Treatment with a Bright Future. J. Neurointerv. Surg..

[27] Won S.-Y., Zagorcic A., Dubinski D., Quick-Weller J., Herrmann E., Seifert V., Konczalla J. (2018). Excellent Accuracy of ABC/2 Volume Formula Compared to Computer-Assisted Volumetric Analysis of Subdural Hematomas. PLoS ONE.

[28] Bechstein M., McDonough R., Fiehler J., Zanolini U., Rai H., Siddiqui A., Shotar E., Rouchaud A., Goyal M., Gellissen S. (2022). Radiological Evaluation Criteria for Chronic Subdural Hematomas: Review of the Literature. Clin. Neuroradiol..

[29] Yamamoto H., Hirashima Y., Hamada H., Hayashi N., Origasa H., Endo S. (2003). Independent Predictors of Recurrence of Chronic Subdural Hematoma: Results of Multivariate Analysis Performed Using a Logistic Regression Model. J. Neurosurg..

[30] Stanišić M., Hald J., Rasmussen I.A., Pripp A.H., Ivanović J., Kolstad F., Sundseth J., Züchner M., Lindegaard K.-F. (2013). Volume and Densities of Chronic Subdural Haematoma Obtained from CT Imaging as Predictors of Postoperative Recurrence: A Prospective Study of 107 Operated Patients. Acta Neurochir..

[31] Edlmann E., Giorgi-Coll S., Whitfield P.C., Carpenter K.L.H., Hutchinson P.J. (2017). Pathophysiology of Chronic Subdural Haematoma: Inflammation, Angiogenesis and Implications for Pharmacotherapy. J. Neuroinflamm..

[32] Lucke-Wold B.P., Turner R.C., Josiah D., Knotts C., Bhatia S. (2016). Do Age and Anticoagulants Affect the Natural History of Acute Subdural Hematomas?. Arch. Emerg. Med. Crit. Care.

[33] Weigel R., Schilling L., Krauss J.K. (2022). The Pathophysiology of Chronic Subdural Hematoma Revisited: Emphasis on Aging Processes as Key Factor. Geroscience.

[34] Holl D.C., Volovici V., Dirven C.M.F., Peul W.C., van Kooten F., Jellema K., van der Gaag N.A., Miah I.P., Kho K.H., den Hertog H.M. (2018). Pathophysiology and Nonsurgical Treatment of Chronic Subdural Hematoma: From Past to Present to Future. World Neurosurg..

[35] Edlmann E., Whitfield P.C., Kolias A., Hutchinson P.J. (2021). Pathogenesis of Chronic Subdural Hematoma: A Cohort Evidencing De Novo and Transformational Origins. J. Neurotrauma.

[36] Chon K.-H., Lee J.-M., Koh E.-J., Choi H.-Y. (2012). Independent Predictors for Recurrence of Chronic Subdural Hematoma. Acta Neurochir..

[37] Miah I.P., Tank Y., Rosendaal F.R., Peul W.C., Dammers R., Lingsma H.F., den Hertog H.M., Jellema K., van der Gaag N.A., Dutch Chronic Subdural Hematoma Research Group (2021). Radiological Prognostic Factors of Chronic Subdural Hematoma Recurrence: A Systematic Review and Meta-Analysis. Neuroradiology.

[38] Uttam B.K., Yuanyuan L., Bizhan A., Thorsten F.R., Mazhar K., Marco C., Dheeraj G. (2023). Short-Term Follow-up Pilot Study of Sole Middle Meningeal Artery Embolization for Chronic Subdural Hematoma: Influence of Internal Architecture on the Radiological Outcomes. Neuroradiology.

